# Subungual squamous cell carcinoma*

**DOI:** 10.1590/abd1806-4841.20165084

**Published:** 2016

**Authors:** Carolina Barbosa de Sousa Padilha, Laila Klotz de Almeida Balassiano, Julyana Calegari Pinto, Flávia Crespo Schueler de Souza, Bernard Kawa Kac, Curt Mafra Treu

**Affiliations:** 1 Policlínica Geral do Rio de Janeiro (PGRJ) – Rio de Janeiro (RJ), Brazil

**Keywords:** Carcinoma, squamous cell, Nails, Nail diseases

## Abstract

Although subungual squamous cell carcinoma is rare, it is the most common primary malignant neoplasms in this location. The higher incidence occurs in the fingernails, but involvement of the toenails is also possible. Subungual squamous cell carcinoma often looks like other more common benign lesions, such as fungal infection, onychomycosis, or viral wart. These factors, together with a general lack of awareness of this disease among physicians, often result in delayed diagnosis. Therefore, it is underdiagnosed, with few reports in the literature. The authors present a case of a man with a diagnosis of subungual squamous cell carcinoma in the hallux, without bone involvement, which was submitted to the appropriate surgical treatment.

## INTRODUCTION

Subungual squamous cell carcinoma (SCC) is a rare cancer that can affect one or more dactyls, with its greater incidence in fingers, but with few cases reported in toes. The tumor usually occurs in middle-aged Caucasian men. The etiology is not fully established, however, some cases have been associated with human papilloma virus (HPV), especially type 16. Other possible causes include chronic trauma, chronic inflammation, exposure to ionizing and/or solar radiation and arsenic. Such associations apply mainly to injuries in the nail bed of the hands, but the etiology for lesions in the nail bed of the feet remains unknown being, most likely, trauma and chronic inflammations.^1^ Diagnosis is difficult and often late. Its atypical clinic picture may mimic other diseases such as verruca vulgaris, onychomycosis, pyogenic granuloma, nail dystrophy, exostosis, chronic paronychia, psoriasis, melanoma and keratoacanthoma. Generally, the suspicion of cancer increases after failure in therapy with antifungal and antibiotics, which may delay and lead to the diagnosis of the disease in its invasive form. Therefore, the objective of this article is to report the case of a patient with subungual SCC with long evolution and alert about the importance of performing early biopsy for histopathological confirmation and therapeutic setting, which will depend on the extent of the tumor. ^2,3^

## CASE REPORT

**Figure 1:**
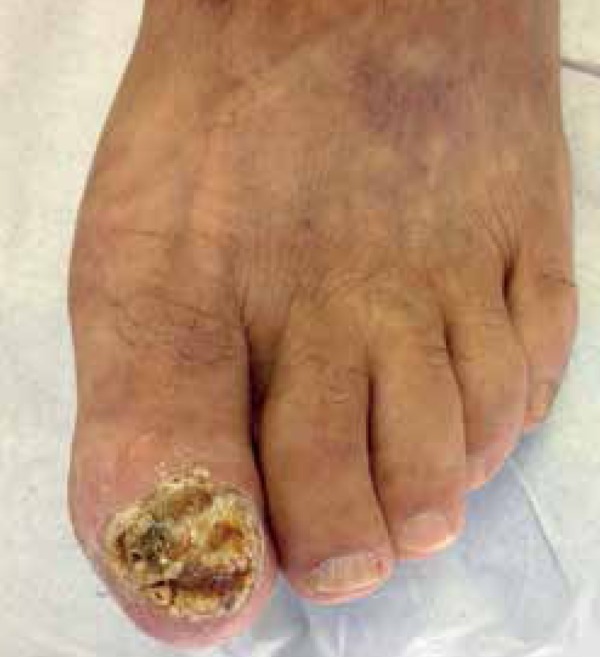
Total destruction of the nail apparatus with serosanguineous discharge

**Figure 2:**
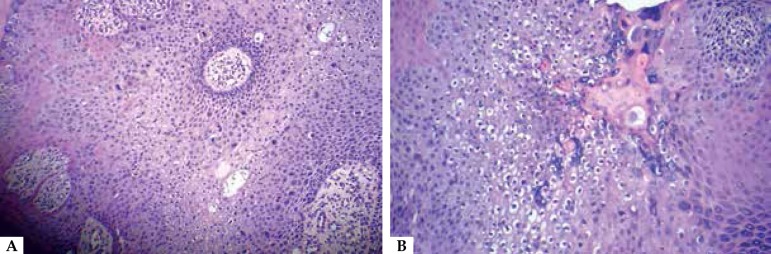
**A**) HE 100X. Neoplasia of atypical pleomorphic keratinocytes with loss of polarity and frequent irregular mitosis; **B**) HE 400X. Focal perinuclear vacuolization

**Figure 3:**
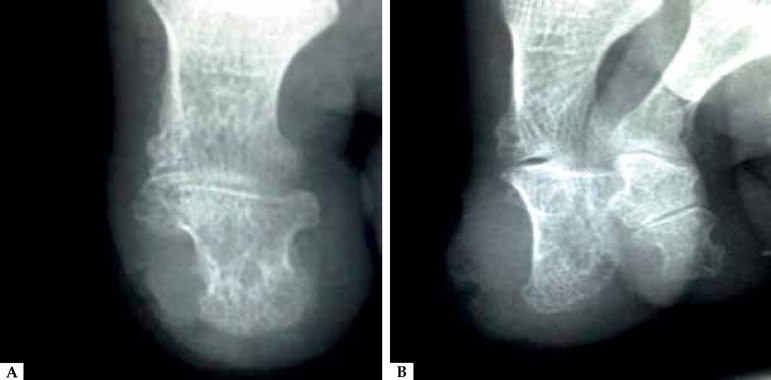
**A**) AP X-ray: No bone involvement; **B**) X-ray Profile: No bone involvement

**Figure 4:**
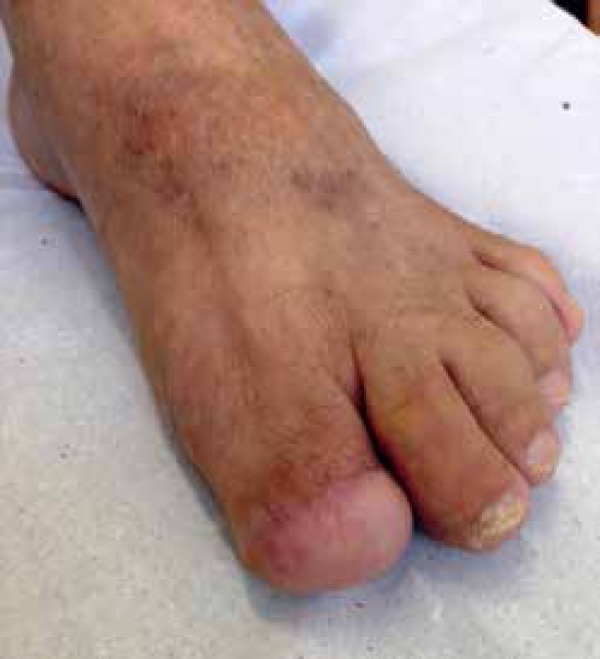
After 3 months of surgery

Man, 75 years old, Caucasian, with history of emergence of nail lesion in the left hallux for 3 years. He had already undergone several treatments with topical and oral antifungal and antibacterial without improvement. He presented pain only during the handling of the lesion. Medical history had no particularities. He was not in use of any systemic medication. He denied trauma, previous skin changes (such as warts or psoriasis), immunosuppression or exposure to radiation or chemical substances. Physical examination showed keratotic and eroded lesions in the nail bed of the left hallux with onychodystrophy, subungual keratosis and serosanguineous discharge (Figure 1). There were no palpable lymph nodes. The initial diagnostic hypotheses were epidermoid carcinoma, keratoacanthoma and subungual wart. A wedge biopsy was performed and the histopathological examination revealed that it was a cancer of atypical pleomorphic keratinocytes with loss of polarity and frequent irregular mitosis confirming our diagnosis of squamous cell carcinoma (Figure 2). Due to the intense perinuclear vacuolation seen in the histopathological examination, we conducted research in DNA for human papilloma virus (HPV) by in situ hybridization technique, however this was negative, discarding it as the causative agent of lesion. A radiograph of the left hallux showed no bone involvement (Figure 3). The patient was then referred to the National Cancer Institute José Alencar Gomes da Silva (INCA) for surgical resolution of the case, where the option was for the distal interphalangeal disarticulation associated with local flap (Figure 4).

## DISCUSSION

Primary subungual squamous cell carcinoma (SCC) in toe is rarely reported in the literature and most of the cases are described in fingernails. Men after the fifth decade of life are statistically most affected. The tumor may manifest associated with lesions of paronychia, onychocryptosis and may lead to dischromia of the nail plate, bleeding and pain. The diagnosis of cancer of the nail bed is usually late because the lesion is confused with benign and infectious diseases.^2^ For this reason it is important to perform biopsy of all the wounds that are persistent and do not heal in the nail bed so early diagnosis, appropriate treatment and follow-up can be done, while the tumor is still confined to its primary site.^4^

Unlike Bowen disease and SCC of the hand, the etiological role of HPV in the development of subungual SCC of the feet is not well established.^4^ In our patient the hybridization in situ test for research in DNA for HPV was negative, which is in agreement with the current literature data.^3^ Both cases of Bowen disease and SCC of the hands associated with HPV showed an important role for oncogenic types found in anogenital mucosa, being the most common the types 16, 33, 51 and 73, suggesting a genital-digital transmission, especially in immunocompromised individuals.^5,6^

Being a cancer rarely detected, subungual SCC does not have a standardized therapeutic approach. The treatment is preferably surgical and depends on the extent of the tumor. Surgical excision with a margin of 5 mm of the total nail apparatus and posterior cover with a skin graft is a good option for subungual SCC in situ, since the partial surgical excision of the nail apparatus is associated with higher recurrence rates and greater discomfort to the patient. Often, amputation or interphalangeal disarticulation is necessary, especially when there is bone involvement.^1,5,7^ In our case despite the absence of bone involvement, the option was for dislocation of the distal phalanx and local flap. Mohs micrographic surgery is a good option and should be encouraged despite the technique difficulty due to the peculiar anatomical unit and histological characteristics of the region.^2,4,5^ Despite the Mohs surgery is the most effective treatment there are reports of 20% recurrence rate for subungual SCC compared with 3% of recurrence for other epidermoid cutaneous carcinomas. Radiation therapy is a treatment option for patients with multiple comorbidities who are not able to tolerate the surgical procedure.^1,8,9^

Bone involvement may occur in 20% of patients. Only 3 cases of death from subungual SCC have been described in the literature.^2,8^ Sentinel lymph node biopsy usually is not part of the surgical algorithm for subungual disease, but reports of inguinal lymph node involvement have been described. Metastasis to lymph node may occur years after treatment of the primary lesion making prognosis extremely poor, with recommended lymphadenectomy or radiotherapy. ^1,10^

This report presented aspects that attracted attention because it is a rare case of subungual SCC in toe, with a typical history of late diagnosis for multiple treatments as benign pathologies that, despite the delay in diagnosis, has been showing good progress after the appropriate surgical treatment.
